# 1555. Evaluating Pre-exposure Prophylaxis Use in a Large Integrated Rural US Health System

**DOI:** 10.1093/ofid/ofad500.1390

**Published:** 2023-11-27

**Authors:** Nikko Rowe A Tabliago, Robert B Lowe, Swathi Gowtham, Darrell McBride

**Affiliations:** Geisinger Medical Center, Johns Island, South Carolina; Geisinger Health System, Danville, Pennsylvania; Geisinger Medical Center, Johns Island, South Carolina; Geisinger Medical Center, Johns Island, South Carolina

## Abstract

**Background:**

The purpose of this analysis was to evaluate pre-exposure prophylaxis (PrEP) use in high-risk patients across a rural health system to establish system-wide interventions for target clinics to improve providers’ knowledge and comfort regarding HIV screening and PrEP provision.

**Methods:**

Retrospective database review was done on total sample of 202,169 participants between the ages of 15 and 40 years, who were seen by a primary care provider (PCP) between November 1st, 2017 and December 31st, 2021. 201,423 participants met inclusion criteria as participants with active HIV and Hepatitis B infections were excluded. High-risk was defined as meeting any of the following criteria: behavioral attributes [multiple partners, men who have sex with men (MSM), and IV drug use], medication use (including prescriptions of ceftriaxone, penicillin and/or azithromycin for STI treatment), positive sexually transmitted infection (STI) test (syphilis, gonorrhea, chlamydia, and trichomoniasis), tested for STIs more than twice and/or tested for HIV more than twice.

**Results:**

Amongst the 201,423 patients, 13.4% (n = 26,972) of the population were identified as high-risk and 174,451 were not considered high-risk. Of the 26,972 patients, only 205 (0.76%) received a PrEP prescription. Those who met the high-risk criteria, were more likely to be female, Black or African American by race, and Hispanic or Latinx by ethnicity. Of those who met the high-risk criteria for PrEP and who received the prescription, they were more likely to be male, MSM, had multiple sexual partners, those that were tested for hepatitis C, hepatitis B, syphilis and HIV infections. Only 2 patients who received PrEP used IV drugs.

**Figure:**
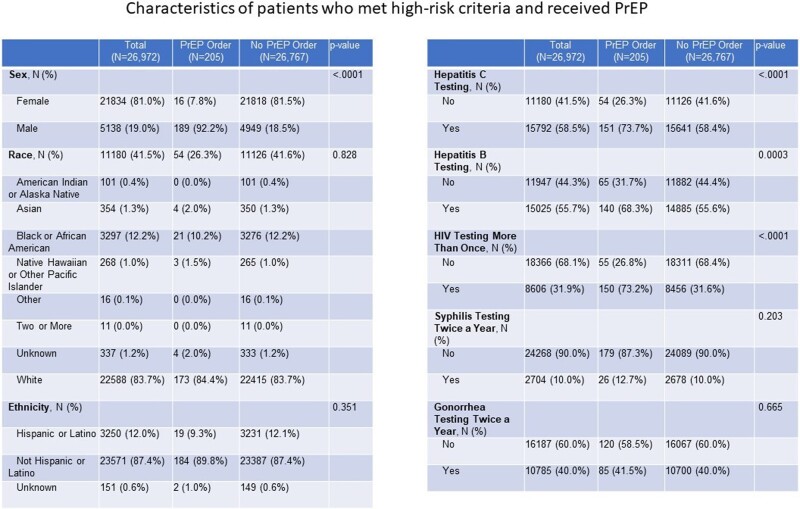

**Figure:**
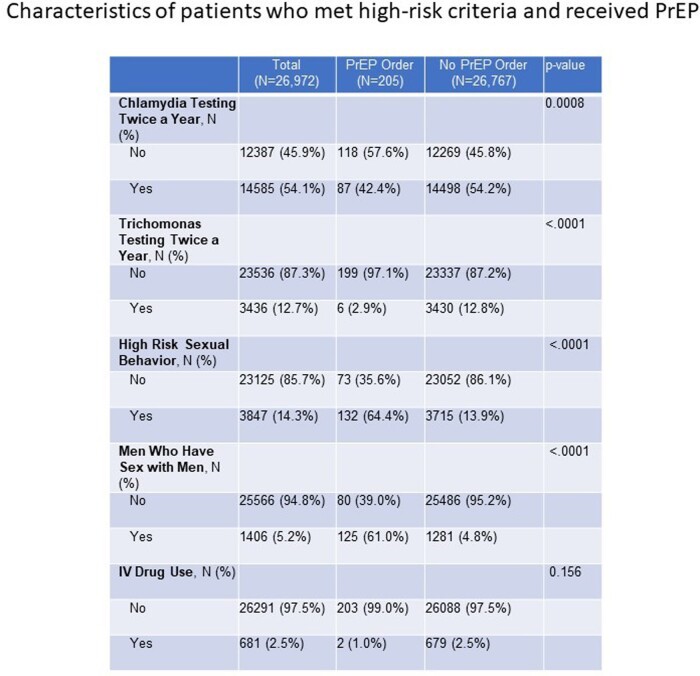

**Figure:**
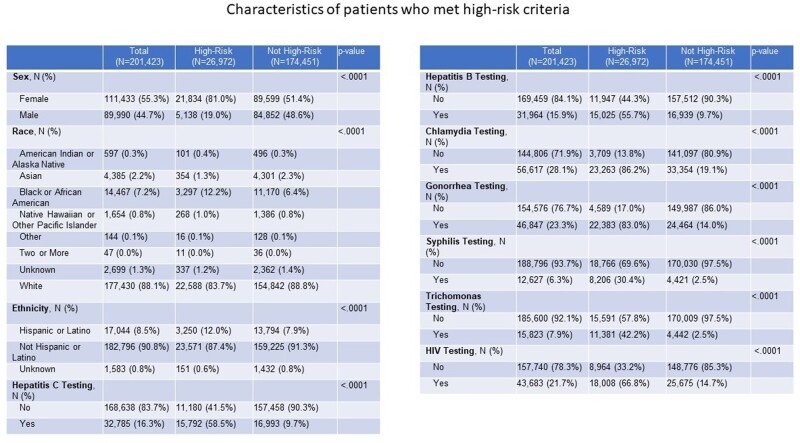

**Conclusion:**

Evaluation of the prescribing practices of PCPs in a large integrated rural health system revealed a large opportunity for meaningful change as only 0.76% of those who met high-risk criteria for HIV acquisition were prescribed PrEP. This study shows that in the setting of a PrEP desert, much needs to be done in the education of providers and communities regarding PrEP use. Unless these urban-rural disparities in PrEP use are addressed, ending the HIV epidemic initiative in the US will not be successful.

**Disclosures:**

**All Authors**: No reported disclosures

